# The Crosstalk Between Osteodifferentiating Stem Cells and Endothelial Cells Promotes Angiogenesis and Bone Formation

**DOI:** 10.3389/fphys.2019.01291

**Published:** 2019-10-14

**Authors:** Tullio Genova, Sara Petrillo, Elisa Zicola, Ilaria Roato, Riccardo Ferracini, Emanuela Tolosano, Fiorella Altruda, Stefano Carossa, Federico Mussano, Luca Munaron

**Affiliations:** ^1^Department of Life Sciences and Systems Biology, UNITO, Turin, Italy; ^2^Department of Surgical Sciences, CIR Dental School, UNITO, Turin, Italy; ^3^Department of Molecular Biotechnology and Health Sciences, UNITO, Turin, Italy; ^4^Department of Clinical and Biological Sciences, UNITO, Orbassano, Italy; ^5^Center for Research and Medical Studies, A.O.U. Città della Salute e della Scienza, Turin, Italy; ^6^Department of Surgical Sciences (DISC), Orthopaedic Clinic-IRCCS A.O.U. San Martino, Genoa, Italy

**Keywords:** mesenchymal stem cells, adipose-derived stem cells, bone vascularization, endothelial cells, co-culture, 3D cultures

## Abstract

The synergistic crosstalk between osteodifferentiating stem cells and endothelial cells (ECs) gained the deserved consideration, shedding light on the role of angiogenesis for bone formation and healing. A deep understanding of the molecular basis underlying the mutual influence of mesenchymal stem cells (MSCs) and ECs in the osteogenic process may help improve greatly bone regeneration. Here, the authors demonstrated that osteodifferentiating MSCs co-cultured with ECs promote angiogenesis and ECs recruitment. Moreover, through the use of 3D co-culture systems, we showed that ECs are in turn able to further stimulate the osteodifferentiation of MSCs, thus enhancing bone production. These findings highlighted the existence of a virtuous loop between MSCs and ECs that is central to the osteogenic process. Unraveling the molecular mechanisms governing the functional interaction MSCs and ECs holds great potential in the field of regenerative medicine.

## Background

Bone formation and remodeling require different cell populations to cooperate so as to regenerate a complex 3D tissue under the guidance of chemical and mechanical cues (Genova et al., 2016). Angiogenesis, the formation of new blood vessels from existing ones, is essential for bone formation and remodeling. The progressive substitution of an avascular cartilage template by a highly vascularized bone tissue is the characterizing feature of endochondral ossification. Likewise, during the fracture healing, which was classically thought to recapitulate the ontogenesis of the tissue (Urist, 1997), angiogenesis precedes osteogenesis and determines its efficiency. The crucial role of vascularization in bone tissue emerges also experimentally from the analysis of genetic models with bone defects, which have indeed been associated to defective vascularization (Schipani et al., 1997; Vu et al., 1998). Achieving the desired synergistic crosstalk between osteoblast precursors and endothelial cells is also crucial for implementing bone tissue engineered therapies based on the mutual influence of osteogenic and angiogenic cues.

Thus, ensuring proper vascularization is mandatory for bone formation, remodeling, and reconstruction (Correia et al., 2011; Genova et al., 2016). Notwithstanding the acknowledged centrality of angiogenesis in bone development, as well as in graft survival after implantation (Griffith and Naughton, 2002), the molecular mechanisms driving vascularization in bone formation and remodeling are still poorly elucidated (Schipani et al., 2009). Thus, a better comprehension of the molecular physiology underlining the crosstalk between osteodifferentiating stem cells and endothelial cell should be sought.

A robust scientific evidence supports that endothelial cells enhance the osteogenic differentiation of mesenchymal stem cells (MSCs) *in vitro* and *in vivo* (Guillotin et al., 2004; Leszczynska et al., 2012; Zhang et al., 2012; Liu et al., 2013). According to equally compelling findings, murine periosteal-derived MSCs promote survival and proliferation of human umbilical vein endothelial cells (HUVECs) through the production of VEGF *in vitro* and enhance *in vivo* vasculogenesis by resuming a pericyte-like phenotype able to support blood vessels (Van Gastel et al., 2012).

In this work, adipose-derived stem cells (ASCs) (Zuk et al., 2001, 2002) have been used as a valuable model to investigate osteodifferentiation. Indeed, ASCs represent an abundant source of MSCs that are easily accessible and can be induced to differentiate in osteoblasts. Consistently, ASCs have been employed successfully to colonize bone graft before their surgical placement, in clinical protocols (Dufrane, 2017). The aim of the present study was to evaluate the functional biological effects of the interaction between osteodifferentiating ASCs and human microvascular endothelial cells (HMECs) as well as to unveil the complex crosstalk mechanisms that take place in the co-culture context.

## Materials and Methods

### Cell Culture

Two cell types were used: human microvascular dermal endothelial cells (HMECs) and adipose-derived stem cells (ASCs).

HMECs were purchased from Lonza (Lonza, Switzerland) and were grown in complete EndoGRO-MV (Millipore, Italy) supplemented with 50 μg/ml gentamicin (Cambrex).

ASCs were isolated from patients who underwent orthopedic treatment with adipose tissue for knee osteoarthritis and signed informed consent, according to the Local Independent Ethics Committee permission (IRB), as previously described (Roato et al., 2019). Briefly, adipose tissue was digested with collagenase NB4 (SERVA Electrophoresis), subsequently washed with saline solution, treated with a cell lysis solution (Promega) to discard blood cells, and then cells were collected and counted. The purity of isolated ASCs was evaluated by flow cytometry, soon after isolation (Zuk et al., 2001; Roato et al., 2018).

### Flow Cytometry Analysis of Mesenchymal Stem Cells Phenotype

ASCs were maintained in culture for two passages, then the following staining procedure was performed with monoclonal antibodies (moAb) fluorocrome-coniugated and isotypic controls: human CD105 PE (Invitrogen), CD73 FITC (kindly provided by Prof. Malavasi, University of Turin), CD44 FITC, CD45 PerCP, IgG1 PE, IgG1 APC and IgG2a PerCP (Miltenyi Biotech), CD90 PerCP (Biolegend), and IgG1 FITC (Immunostep). About 10^5^ events/sample were used for capture with CellQuest software. Data were analyzed with Flowlogic software (Miltenyi Biotec).

To evaluate the morphology, cells were stained with Rhodamine-Phalloidin and DAPI after incubation in PBS containing 0.3% Triton X-100.

### Osteogenic Cell Differentiation

To obtain osteogenic differentiation, 10 × 10^4^/well ASCs were cultured in a six-well plate in osteogenic medium (OM) for 7 days by supplementing the normal growth medium with 10 mM β-glycerophosphate, 50 μg/ml ascorbic acid, and 0.02 mg/ml dexamethasone. Prior to experiment, dexamethasone was removed in order to avoid any inhibitor effect on endothelial cells as reported in literature (Mussano et al., 2017a).

### Co-cultures

For co-cultures, transwell inserts 0.4 μm pore PC membrane (Transwell, Corning, USA) were used.

For migration, proliferation and tubulogenesis assays, ASCs (2 × 10^4^ cells/ml) were seeded into 24-well transwell inserts (upper chamber) and HMECs were seeded into 24-well plates (bottom).

For qRT-PCR analysis, 10 × 10^4^ HMECs were seeded into six-well 0.4 μm pore inserts, whereas ASCs were seeded into six-well plates (bottom), thus ensuring proper RNA extraction from ASCs.

### Proliferation

Cells were plated at a density of 2,500 cells/well in 24-well culture dishes, and the proliferation was assessed by cell count and CellTiter-Glo^®^ (Promega, Milan, Italy) according to the manufacturer’s protocol at 1, 3, and 7 days (Canullo et al., 2017b; Mussano et al., 2017b,c). This Luminescent Cell Viability Assay is a homogeneous method of determining the number of viable cells in culture based on quantitation of the ATP present. The amount of ATP is directly proportional to the number of viable cells in culture (as reported by the manufacturer).

### Migration Assay

Cell motility was investigated as migration of cells into a wound introduced in a confluent monolayer. 4 × 10^4^ HMECs were seeded in two chamber cell culture silicone inserts (Ibidi, Germany) for 24 h (after cell attachment, the insert is removed and a cell-free gap is created).

Cells did not undergo any significant degree of mitosis during the experiments. Experiments were done using a Nikon Eclipse Ti-E microscope with a 4X objective. Cells were kept at 37°C and 5% CO_2_ for all experiments, acquisition was obtained through Metamorph software (Molecular Devices, Sunnyvale, California, USA) (Avanzato et al., 2016; Canullo et al., 2016, 2017a). Cell motility into a wound was measured with Metamorph software and was expressed as percentage of cell migration (Fiorio Pla et al., 2010, 2014; Basilico et al., 2015). At least three fields for each condition were analyzed in each independent experiment. At least three independent experiments were performed for each experimental condition.

### Chemotaxis Assay

HMECs were seeded into Transwell inserts with 8 μm pore (0.5 × 10^4^/transwell) and maintained in EndroGRO. The following day, transwells with HMECs were put into a 24-well plate (bottom chamber) with ASCs maintained in GM or OM. After 4h, transwell inserts were fixed with 4% paraformaldehyde and stained with DAPI. Non-migrated cells were removed using a cotton swab. Total migrated cells (nuclei) were counted.

### *In vitro* Angiogenesis Assay

*In vitro* formation of capillary-like structures was performed on growth factor-reduced Matrigel (Corning, USA) in 24-well plates. Cells (3.5 × 10^4^ cells/well) were seeded on the Matrigel and maintained in EndoGRO-MV. Cell organization in Matrigel was acquired after 10 h with a Nikon Eclipse Ti E microscope using a Nikon Plan 10X/0.10 objective. At least three independent experiments were done for each experimental condition (Genova et al., 2017).

With ImageJ’s Angiogenesis Analyzer tool of Gilles Carpentier several parameters were analyzed:

1. nodes are pixels with three neighbors represented as a circular dot;
2. junctions correspond to nodes or group of fusing nodes;
3. segments are elements delimited by two junctions;
4. isolated elements are binary lines which are not branched;
5. master segments consist in pieces of tree delimited by two junctions none exclusively implicated with one branch, called master junctions; and
6. master junctions are junctions linking at least three master segments. Optionally, two close master junctions can be fused into a unique master junction.

With the word “tree,” we identify the complex structure made of nodes and segments that endothelial cells form *in vitro* during this experiment.

### RNA Extraction and Real-Time Polymerase Chain Reaction Analysis

Total RNA was extracted using PureLink RNA Mini Kit (Ambion, Life Technologies Italy). For quantitative real-time polymerase chain reaction (qRT-PCR), 1 μg total RNA was transcribed into complementary DNA by MultiScribe^®^ Reverse Transcriptase (High-Capacity cDNA Reverse Transcription Kit, Thermo Fisher Scientific), and PCR analysis was then assessed using TaqMan probes from Roche. Transcript abundance, normalized to 18 s mRNA expression, is expressed as a fold increase over a calibrator sample. qRT-PCR was performed on a 7900HT Fast Real-Time PCR System (Applied Biosystems, Life Technologies Italy) (Herrera Sanchez et al., 2017; Ingoglia et al., 2017; Beneduce et al., 2019). Specific primers and probes were designed using the Universal Probe Library–Assay Design Center–Roche Life Science software^1^.

qRT-PCR primers:

**Table 1:** 

1. *ANG1:*	*gacagatgttgagacccaggta*	*tctctagcttgtaggtggataatgaa*
2. *ANG2:*	*tgcaaatgttcacaaatgctaa*	*aagttggaaggaccacatgc*
3. *VEGF-A:*	*ctacctccaccatgccaagt*	*ccatgaacttcaccacttcgt*
4. *PDGF-β:*	*tgatctccaacgcctgct*	*tcatgttcaggtccaactcg*
5. *TGF-β:*	*actactacgccaaggaggtcac*	*tgcttgaacttgtcatagatttcg*
6. *FGF-2:*	*ttcttcctgcgcatccac*	*ttctgcttgaagttgtagcttgat*
7. *BMP-2:*	*gactgcggtctcctaaaggtc*	*ggaagcagcaacgctagaag*
8. *OPN:*	*gagggcttggttgtcagc*	*caattctcatggtagtgagttttcc*

### Bioreactor and Scaffolds

About 7 mm heterologous cancellous bone matrix disks (Sp-Block, Tecnoss, Italy) were used as bone scaffolds and ASCs and HMEC were seeded (simultaneously in the co-culture condition).

In order to obtain a proper cell growth on bone scaffolds, the LiveBox2 bioreactor (IVTech, Italy) was used (Cacopardo et al., 2019). The bioreactor is composed of a peristaltic pump, a reservoir, and a perfusion chamber. The perfusion chamber (which is composed of two chambers separated by a porous membrane) was used in a sigmoidal flux mode. This configuration determines a cell culture media flux from the upper chamber to the lower chamber, allowing a proper scaffold perfusion. The bioreactor was kept under a humidified atmosphere of 5% CO_2_ in air, at 37°C using a 0.5 ml/min flux.

### X-Ray Microtomography

Bone scaffolds were analyzed by high-resolution X-ray microtomography (SkyScan 1172, Bruker) to study the new bone formation as described in a previous work (Roato et al., 2018). (80 kV using a 0.5 mm Al filter at a resolution of 6 μm, 0.4° of rotation step, 360° scan, and 4X frame averaging). A color contrast mask was used to allow a clear identification of newly formed mineralized tissue. The quantification of bone was performed by measuring the mineralized tissue length by using DataViewer software (Bruker).

### Statistical Analysis

*In vitro* angiogenesis images were analyzed with the plug-in Angiogenesis Analyzer of ImageJ (Wayne Rasband, NIH, U.S.A.).

Data were analyzed by GraphPad Prism7 (GraphPad Software, Inc., La Jolla, USA) and Microsoft Excel. Each experiment was repeated at least three times. Statistical analysis was performed by using the Mann-Whitney non-parametric test or two-way ANOVA with Tukey’s multiple comparisons test. A *p* <0.05 was considered significant.

qRT-PCR data were analyzed by performing ordinary two-way ANOVA with Sidak’s multiple comparisons test for grouped analyses or Mann-Whitney test for column analyses.

## Results

### Characterization and Osteodifferentiation of Adipose-Derived Stem Cells

**Figure 1:**
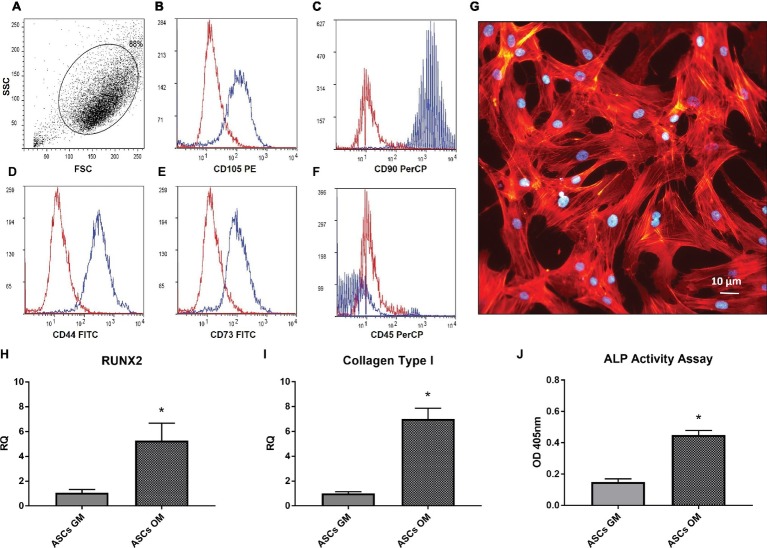
Characterization adipose-derived stem cells. **(A–F)** Flow cytometer analysis performed on *in vitro* cultured ASCs; the expression of CD105, CD44, CD90, CD73 **(B–E)** and the absence of CD45 **(F)** is shown. **(G)** Morphology of isolated ASCs, stained by phalloidin(red) and Dapi (Blue). **(H,I)** Real-time PCR analysis of RUNX2 and Coll-I expression in ASCs after 7 days of culture in osteogenic medium (OM) or growth medium (GM). Data represent mean ± SEM. ^*^p < 0.05. **(J)** Alkaline phosphatase (ALP) activity in ASCs after 7 days of culture in OM or GM.

The purity of the isolated ASCs was assessed through flow cytometry analysis. In Figure 1A, a homogeneous cell population expressing mesenchymal markers is evident, showing a high expression of cells positive for CD105, CD44, CD90, CD73 and negative for CD45 (Figures 1B–F). The morphology of ASCs (Figure 1G) well resembled the typical one of other stem cells obtained, e.g., from bone marrow or umbilical cord blood. Successively, we investigated the ability of ASCs to undergo osteodifferentiation in culture with the osteodifferentiating medium (OM). As shown in Figures 1H,I, the OM enhanced the production of Runt-related transcription factor 2 (RUNX2) and Collagen type 1, key markers of osteodifferentiating cells. Consistently, after 7 days of osteodifferentiation, also the level of Alkaline phosphatase (ALP) activity was significantly increased compared to undifferentiated ASCs (Figure 1J).

### Osteodifferentiating Adipose-Derived Stem Cells Promote Proliferation, Migration, and Vessel Recruitment of Human Microvascular Endothelium

**Figure 2:**
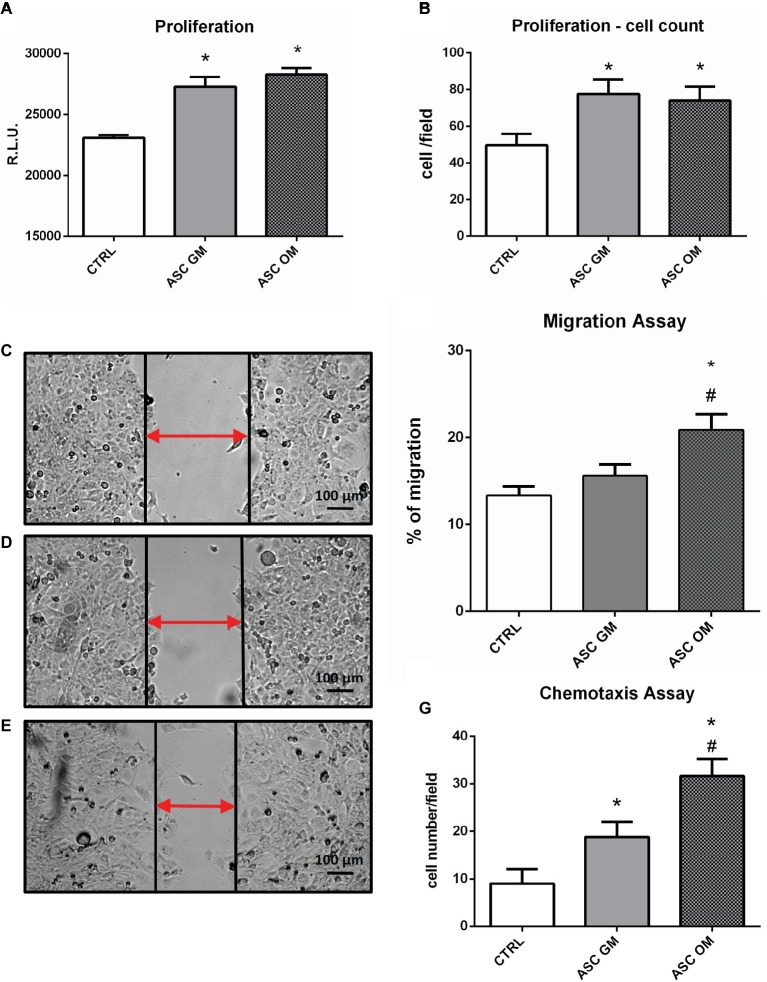
Biological effects induced by ASCs on co-cultured endothelial cells. **(A)** ATP-based proliferation assay performed on HMECs upon 24 h of co-culture with ASCs. **(B)** Cell count (DAPI staining) proliferation assay. **(C–F)** Migration assay performed on HMECs alone (CTRL) or upon co-culture with ASCs maintained in growth medium (GM) or osteodifferentiating medium (OM). **(C–E)** Representative picture of wound healing assay. **(F)** Quantification of endothelial cells migration in the indicated co-culture conditions. Data represent migration rate of HMEC after 8 h. **(G)** Chemotaxis assay performed on HMECs. The symbol (*) indicates statistical significance of ASC OM or ASC GM compared to the control condition (without ASCs) considering a *p* <0.05. The symbol (#) indicates statistical significance of ASC OM compared to ASC GM considering a *p* <0.05. CTRL, HMEC without ASCs; ASC GM, HMEC in co-culture with undifferentiated ASC; ASC OM, HMEC in co-culture with osteodifferentiating ASC.

In order to evaluate the biological effects of osteofferentiating ASCs on co-cultured endothelial cells, an ATP-based proliferation assay and a cell count were performed after 24 h of co-culture. As shown in Figures 2A,B, the presence of ASCs significantly increased the level of HMECs proliferation, regardless of osteodifferentiation. Next, the ability of ASCs to recruit microvascular endothelium was tested by performing a chemotaxis-based assay. Osteodifferentiating ASCs (Figure 2G) showed a significantly increased ability to attract endothelial cells when compared to undifferentiated ASCs. Endothelial cell migration is an essential step in bone tissue remodeling and vascularization. To understand how the differentiation of ASCs affects endothelial migration, a co-culture migration assay was performed in the presence of HMECs. Endothelial cells displayed a significantly enhanced migratory potential when co-cultured with osteodifferentiating ASCs. Conversely, undifferentiated ASCs did not affect HMEC migration (Figures 2C–F).

### Osteodifferentiating Adipose-Derived Stem Cells Increase *in vitro* Angiogenesis

**Figure 3:**
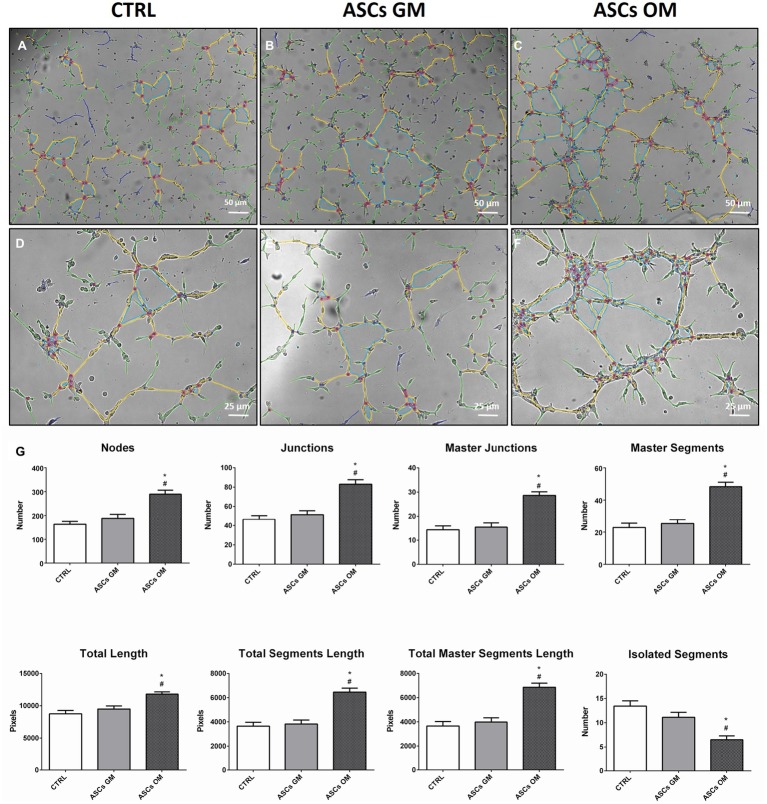
Evaluation of the angiogenic potential of HMECs in the presence of ASCs. **(A–F)** Representative pictures of *in vitro* tubulogenesis assay performed on endothelial cells alone (CTRL) or upon co-culture with ASCs. The formation of capillary-like structures was performed on Matrigel coating and analyzed after 8 h using ImageJ’s Angiogenesis Analyzer tool. The quantification of main parameters describing the capillary network complexity is reported in **(G)** CTRL, HMECs without ASCs; ASCs GM, HMECs co-cultured with undifferentiated ASCs; ASCs OM, HMECs co-cultured with osteodifferentiating ASCs. The symbol (*) indicates a statistical significance vs. the CTRL condition; the symbol (#) indicates a statistical significance of the ASCs GM condition compared to ASCs OM condition, considering a *p* < 0.05.

During bone tissue formation, vascular endothelium migrates and forms a network of small capillaries to provide oxygen and nutrients to the newly formed tissue. For this reason, *in vitro* HMEC angiogenesis was evaluated to understand how this process is modulated by ASCs (Figure 3). HMECs were co-cultured with either osteodifferentiating ASCs or non-osteoinduced ASCs, in a tubulogenesis assay *in vitro*. A complete characterization of the complexity of the newly formed capillary network (in terms of number of nodes, number of master junctions, number of segments, total length of the newly formed “tree,” total segments length, and total master segments length) is shown in Figure 3. The first three parameters represent the complexity of the interconnections inside the network; the last three are a measure of the capillary network extension in space. The capillary network shows higher complexity when HMECs were co-cultured with osteodifferentiating ASCs compared to undifferentiated cells (Figure 3).

### Endothelial Cells Induce the Release of Pro-angiogenic Factors by Adipose-Derived Stem Cells and Promote Their Osteodifferentiation

**Figure 4:**
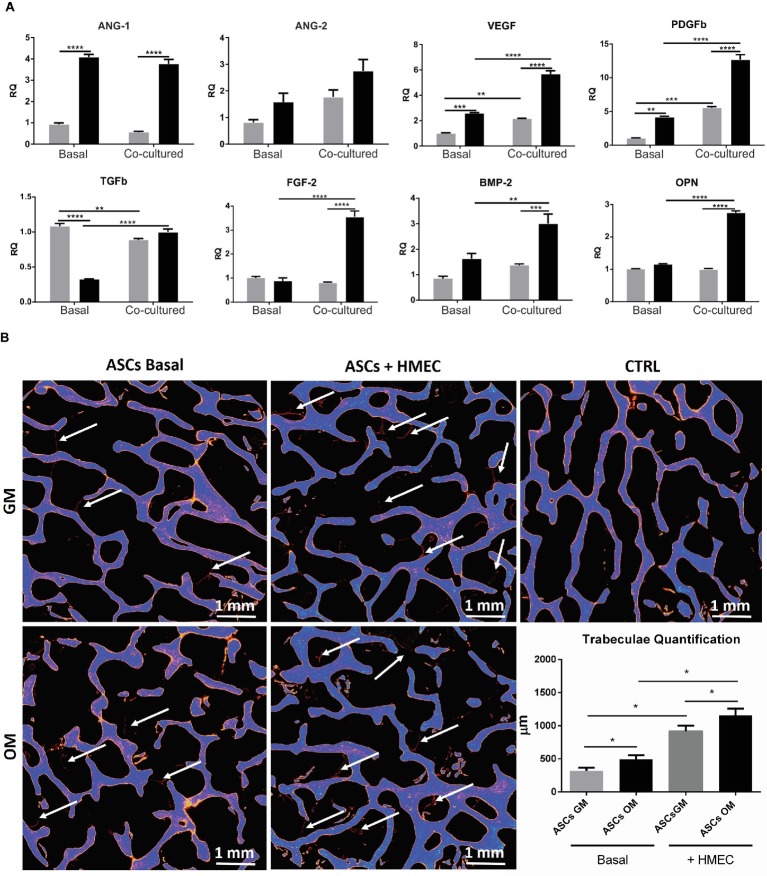
**(A)** Expression profile of growth and angiogenic factors in co-cultured ASCs and HMECs. qRT-PCR analysis of growth and angiogenic factors performed on ASCs under basal conditions (without endothelial cells) or after 4 h of co-culture with HMECs. Undifferentiated (gray bar) and osteodifferentiating (black bar) ASCs are shown. Data represent mean ± SEM; *n* = 3. ^*^p < 0.05; ^**^ < 0.01; ^***^p < 0.001; ^****^p < 0.0001. Statistical analysis: ordinary two-way ANOVA with Sidak’s multiple comparisons test for grouped analyses. Ang1, Angiopoietin 1; Ang2, Angiopoietin 2; VEGF, vascular endothelial growth factor; PDGF-b, platelet-derived growth factor b; TGF-b, transforming growth factor b; FGF-2, basic fibroblast growth factor; BMP-2, bone morphogenetic protein 2; OPN, osteopontin. **(B)** Evaluation of bone formation by X-ray microtomography. Representative images of X-ray microtomography of bone scaffolds. ASCs were seeded in the bone scaffold and maintained in osteodifferentiating medium for 30 days. The experiment was performed in the presence (ASCs + HMEC) or not (ASCs basal) of endothelial cells. The bone scaffold is shown in blue, whereas the newly formed mineralized tissue is in red (indicated by white arrows). Quantification of the newly formed mineralized tissue for each experimental condition is reported in the histogram. ^∗^*p* < 0 05. CTRL, bone scaffold alone; ASCs GM (gray bar) cultured for 7 days in growth medium before being seeded in the bone scaffold; ASCs OM (black bar) cultured for 7 days in osteodifferentiating medium before being seeded in the bone scaffold.

As stated above, osteodifferentiating ASCs can increase the recruitment of endothelial cells and enhance angiogenesis. To investigate the molecular mechanisms underlying these biological effects, we studied the expression profile of pro-angiogenic factors released by osteodifferentiating ASCs compared to undifferentiated ASCs. As shown in Figure 4A, the expression of key pro-angiogenic genes as Angiopoietin 1 (ANG-1), Angiopoietin 2 (ANG-2), vascular endothelial growth factor (VEGF), platelet-derived growth factor b (PDGFb), transforming growth factor b (TGFb), and basic fibroblast growth factor (FGF-2) is higher in osteodifferentiating ASCs (gray bars) compared to undifferentiated ASCs (black bars). These data indicate that osteodifferentiating ASCs release pro-angiogenic factors, thus enhancing angiogenesis.

To investigate whether the presence of endothelial cells may increase the ability of osteodifferentiating ASCs to promote angiogenesis, we performed the same gene expression screening in ASCs upon co-culture with HMECs. Importantly, the presence of endothelial cells can further enhance the production of some pro-angiogenic factors (i.e., VEGF, PDGFb, and FGF2) in osteodifferentiating ASCs compared to the basal condition (without endothelial cells) (Figure 4A).

Finally, we evaluated the effects of the interaction between ASCs and endothelial cells in the process of osteodifferentiation. To this aim, we analyzed the expression of osteogenic markers like bone morphogenetic protein 2 (BMP-2) and osteopontin (OPN) (Figure 4A). Importantly, the presence of endothelial cells can strongly increase the expression of both BMP-2 and OPN in osteodifferentiating ASCs. Taken together, these data highlight a complex crosstalk in which osteodifferentiating ASCs produce higher amount of pro-angiogenic factors, and endothelial cells in turn are able to promote the osteodifferentiation of ASCs.

### Endothelial Cells Increase Bone Production *in vitro*

To test the ability of endothelial cells to stimulate bone formation, we co-cultured HMECs and ASCs in a 3D *in vitro* model. A bone scaffold was maintained in a perfused bioreactor (LB2 IVTech) to obtain a more reliable and physiological environment. To evaluate the bone production within the scaffold, we measured the newly formed trabeculae after 1 month of culture, using micro-tomography (Figure 4B). Notably, we found that the presence of endothelial cells strongly increases bone production by ASCs.

This result supports the crucial role of endothelial cells in the process of osteodifferentiation of ASCs.

## Discussion

A strong crosstalk between osteoprogenitors and the vascular network underlies the processes of bone formation, remodeling, and healing. For this reason, endothelial cell recruitment and organization becomes paramount to all these processes (Carano and Filvaroff, 2003; Jain, 2003; Hankenson et al., 2011). Thus, the co-culture condition was selected as the *in vitro* context closest to the physiological condition and the most suitable to unveil the mutual influence of osteodifferentiating cells and endothelial cells. To induce osteodifferentiation, ASCs were maintained for 7 days in the traditional osteoinducing culture medium (OM) containing dexamethasone. This time window allowed achieving early osteodifferentiation meanwhile avoiding the loss of stemness features in the untreated counterpart. During co-culture experiments, the differentiation medium was deprived of dexamethasone to avoid a negative bias in endothelial cells response possibly deriving from the anti-angiogenic effects of this synthetic glucocorticoid (Heffernan et al., 1978; Mirabelli et al., 2014).

Our data collectively demonstrate that pre-osteodifferentiated ASCs promote endothelial cell recruitment and enhance their functionality, thus providing potential benefits in the clinical outcome.

The numerous advantages deriving from the use of ASCs co-cultures for the treatment of bone defects have already been reported. For instance, ceramic scaffolds containing both ASCs and bone marrow stromal cells (BMSCs) has proved more effective for the treatment of calvarial defects in mice compared to ceramic plus ASCs alone (Kim et al., 2014).

To our knowledge we showed, for the first time, that osteodifferentiating ASCs enhance the response of HMECs in terms of migration, recruitment, and the ability to form capillary-like structures *in vitro*.

To quantify the angiogenic effect induced by the co-culture, some key descriptors of the capillary network complexity were considered, following established protocols (Petrillo et al., 2018). In particular, the number of nodes, master segments, and master junctions may suggest how the network is organized in terms of interconnection and progress toward intensification, which ultimately translates into a better vascularization. On the other hand, the parameter representing the total length of the structure indicates how the network tends to reach deeper into space, which translates into a more functional vascularization, an essential property of bone tissue. On the contrary, the number of isolated segments inversely correlates with a strong and complex vascularization: the higher the number of isolated segments, the weaker the network and the poorer its organization. All these parameters taken together, along with the chemotaxis and migration data, strongly support the enhanced response of endothelial cells due to the influence of osteodifferentiating ASCs compared to the undifferentiated counterpart.

In the present work, we aimed to elucidate the putative paracrine factors responsible for the MSC-endothelial cells crosstalk occurring during osteogenic differentiation and vascular remodeling. Thus, we analyzed the expression of angiogenic and growth factors by ASCs in co-culture with or without endothelial cells. In particular, the mRNA levels of the main angiogenic and growth factors were detected through qRT-PCR in osteodifferentiating ASCs co-cultured with HMECs. We found that osteodifferentiating ASCs produced pro-angiogenic factors responsible for vessels recruitment and angiogenesis. Importantly, the presence of endothelial cells further increased the release of pro-angiogenic factors by ASCs but also promoted the osteodifferentiation of ASCs, thus providing a virtuous loop of interaction.

These findings are in agreement with previous studies on 3D-culture systems, showing that the co-culturing of ASCs with HUVECs increases the production of angiogenesis-related genes in both cell types and that these effects are mediated by the activation of Wnt/β-catenin pathway (Cai et al., 2017).

In the present work, the pro-angiogenic factor VEGF-A was found up-regulated in ASCs upon co-culture with HMECs. The release of VEGF can trigger important responses in both ASCs and endothelial cells through a paracrine stimulation. For instance, VEGF has previously been reported to improve ASCs osteogenic differentiation (Clark et al., 2015). Consistently, the expression level of the osteodifferentiation marker BMP-2 was higher when ASCs were co-cultured with HMECs. Originally described as the main player in the activation of the cascade of endochondral bone formation (Wozney et al., 1988), BMP-2 is now recognized as a multi-purpose cytokine stimulating cell migration (Cunningham et al., 1992; Willette et al., 1999) and both osteoclasts and osteoblasts differentiation (Abe et al., 2010). Notably, the production of pro-angiogenic factors as VEGF-A is actually stimulated by BMPs, particularly BMP-2/-4, as it has been shown in fracture-healing models *in vivo* (Suwa et al., 1998; Ripamonti et al., 2000; Moutsatsos et al., 2001). Here, we showed that enhanced angiogenesis is achieved through the production of osteoblast-derived angiogenic factors and osteodifferentiation is in turn increased by endothelial cells (Wang et al., 1997; Deckers et al., 2002). To further demonstrate the pivotal role of paracrine stimuli in the crosstalk between ASCs and ECs, it would be of primary importance to directly measure the angiogenic and growth factors released by both cell types.

To overcome the classical limitations of traditional 2D culture systems, we seeded ASCs in 3D bone scaffolds kept in a perfused millifluidic bioreactor. This model further supported the crucial role of endothelial cells in the process of bone formation. Indeed, the formation of bone trabeculae was strongly enhanced when ASCs where seeded in bone scaffolds in the presence of endothelial cells.

In recent years, great efforts have been made to improve bone regeneration techniques, owing not only to the technological innovations in the field of material science but also to a growing variety of sources of stem cells for autologous transplants (Derubeis and Cancedda, 2004; Correia et al., 2014).

Among all the possible choices, MSCs hold great potential for bone tissue engineering (Samsonraj et al., 2017). In the present work, we focused our attention on ASCs, which can be easily isolated from the adipose tissue with very low risks of infection or pain (Mussano et al., 2018).

The suitability of stem cells in bone tissue engineering depends both on their ability to differentiate into osteoblasts and on their paracrine effects (Frontiers in bioengineering and biotechnology). Nowadays, stem cells are mostly implanted as undifferentiated cells and bone-inducing factors are provided in loco to drive the differentiation process (Chen et al., 2019). This therapeutic approach, however, is not entirely free of risks (Skovrlj et al., 2015).

Here, we compared the biological response of undifferentiated ASCs to that of pre-differentiated ASCs kept in OM for 7 days, this time period of induction being the shortest capable to induce an early osteodifferentiation (Sabatino et al., 2012; Hanley et al., 2014). This study highlights the prevalent role of osteodifferentiating ASCs, compared to the less differentiated cells of the same lineage, in promoting endothelial remodeling required for efficient vascularization. Notably, osteodifferentiating ASCs trigger strong angiogenic responses on microvascular endothelial cells in terms of migration, recruitment and tubule formation. This evidence may support the use of *ex vivo* differentiated ASCs to accelerate bone regeneration in tissue engineering. To overcome the intrinsic limitations of *in vitro* studies, the next step would be the use of scaffolds containing both ASCs and endothelial cells in pre-clinical models of bone defects in order to provide evidences of the clinical benefits deriving from these findings. In future studies, differentiated ASCs could be injected *in vivo* to test their osteoblast identity and their actual ability to be integrated in the host tissue without the risk of rejection or bone formation defects.

## Data Availability Statement

All the dataset are available at Department of Life Sciences and Systems Biology, UNITO Italy. tullio.genova@unito.it.

## Author Contributions

TG and SP performed experimental procedures, data analysis, experimental designing, and manuscript writing. EZ performed experimental procedures and data analysis. IR performed experimental procedures and data analysis and provided scientific support. RF performed data analysis and provided scientific support. ET provided scientific support and helped in manuscript writing. FA provided scientific support and helped in manuscript writing. SC provided scientific support. FM and LM performed data analysis, experimental design, provided scientific support, and helped in manuscript writing.

### Conflict of Interest

The authors declare that the research was conducted in the absence of any commercial or financial relationships that could be construed as a potential conflict of interest.

## Footnotes

^1^www.lifescience.roche.com
